# E-cigarette or Vaping Product Associated Lung Injury (EVALI) Presenting As Cardiac Arrest

**DOI:** 10.7759/cureus.25010

**Published:** 2022-05-15

**Authors:** Natalia Schekochikhina, Riley Meister, Kovid Trivedi

**Affiliations:** 1 Internal Medicine, Western University of Health Sciences, Lebanon, USA; 2 Pulmonary/Critical Care Medicine, Salem Pulmonary Associates, Salem, USA

**Keywords:** e-smoking, vaping, e-cigarettes, electronic cigarettes, cardiac arrest, corticosteroid treatment, vitamin e, public and environmental health, out of hospital cardiac arrest, e-cigarette and vaping product use associated lung injury (evali)

## Abstract

E-cigarettes or vaping products became available in the market in 2004. Since then, their use has rapidly increased in all sections of society. They have been increasingly used as a "safer" alternative for combustible cigarettes and as an aid toward smoking cessation. Over time, the acceptability of e-cigarettes in public spaces increased. Lack of regulatory control also led to a rapid rise in the rate of e-cigarette/vaping product users. We report a case of a 35-year-old female who recently switched from conventional cigarettes to e-cigarette usage, and who presented to the emergency department after an out-of-hospital cardiac arrest. She was found to have bilateral extensive nodular ground-glass opacities on a CT angiogram of the chest. She needed non-invasive ventilation and was initially started on broad-spectrum antibiotic treatment for possible pneumonia. Due to a worsening clinical status, e-cigarette or vaping product associated lung injury (EVALI) diagnosis was considered, and she was started on parenteral steroid therapy, leading to rapid recovery in respiratory status. With a tapering course of steroid therapy and cessation of e-cigarette use, there was complete clinical and radiological recovery. This case highlights that EVALI can have varied clinical presentations, and the diagnosis should be considered in anyone who presents with an acute cardio-pulmonary decline and a concomitant history of e-cigarette use.

## Introduction

Since their market availability in 2004, the prevalence of e-cigarette use and vaping has increased dramatically, spurred in part by claims that these smoking-alternative devices enable individuals to inhale non-toxic aerosolized vapors. Additionally, e-cigarettes are touted as means of smoking cessation and are more commonly accepted in public spaces [1]. The 2018-2019 Tobacco Use Supplement to the Current Population Survey, the largest in the U.S., estimates that 5.66 million adults currently use e-cigarettes [2]. This is problematic, as evidence is mounting to support that contrary to their initial promise, these devices can be addictive, irritating, toxic, and carcinogenic [3]. Several pathologies have been described with linkage to e-cigarettes that include explosion-induced trauma and asthma amongst other serious adverse events [4, 5]. Many of these pathological phenomena are grouped into a category known as e-cigarette or vaping product associated lung injuries (EVALI), which is considered a diagnosis of exclusion for individuals with a smoking history [6]. United States (US) Center for Disease Control (CDC) reports from February 2020 indicated that within the US and its associated territories, there have been over 2800 EVALI hospitalizations or deaths [7]. While investigations are ongoing to identify the causative mechanisms of EVALI injuries, vitamin E acetate was shown to have a significant association, eventually leading to the removal of the chemical from marketed products. [7]. This case report describes a young female with a history of e-cigarette use who had an out-of-hospital cardiac arrest as a presentation for EVALI.

## Case presentation

A 35-year-old female with a history of anxiety was brought into the emergency department (ED) following an out-of-hospital cardiac arrest. The patient became unresponsive at home, was pulseless, and not breathing; witnessing this, the husband started chest compressions and called 911. Based on the 911 operator’s instructions, the husband continued cardiopulmonary resuscitation (CPR) for about 15 minutes. Paramedics continued CPR for about two more minutes before the return of spontaneous circulation (ROSC). Within the next few minutes, she became more responsive and had spontaneous breathing. Saturation was 60% on room air. She was started on supplemental oxygen via nasal cannula by the paramedics and brought to the ED for further evaluation. The patient admitted to having nausea and vomiting for the last three to four days and denied any respiratory or other complaints prior to losing consciousness. She complained of chest wall pain after receiving chest compressions. She had been smoking conventional cigarettes for the last 11 years and recently had switched to e-cigarettes. She has a history of alcohol, marijuana, methamphetamine, cocaine, and psychedelic mushroom abuse. Currently, she only uses inhalational tetrahydrocannabinol (THC) intermittently.

On examination, the patient was ill-appearing, tachycardic, in acute respiratory distress, and had bilateral diffuse crackles on chest exam. The rest of the exam was unremarkable. Vitals included heart rate of 112/min, blood pressure (BP) of 145/88 mm Hg, afebrile, and saturation of 97% on 4L/min supplemental oxygen. She tested negative for COVID-19 a day ago at her primary care physician’s office.

**Figure 1:**
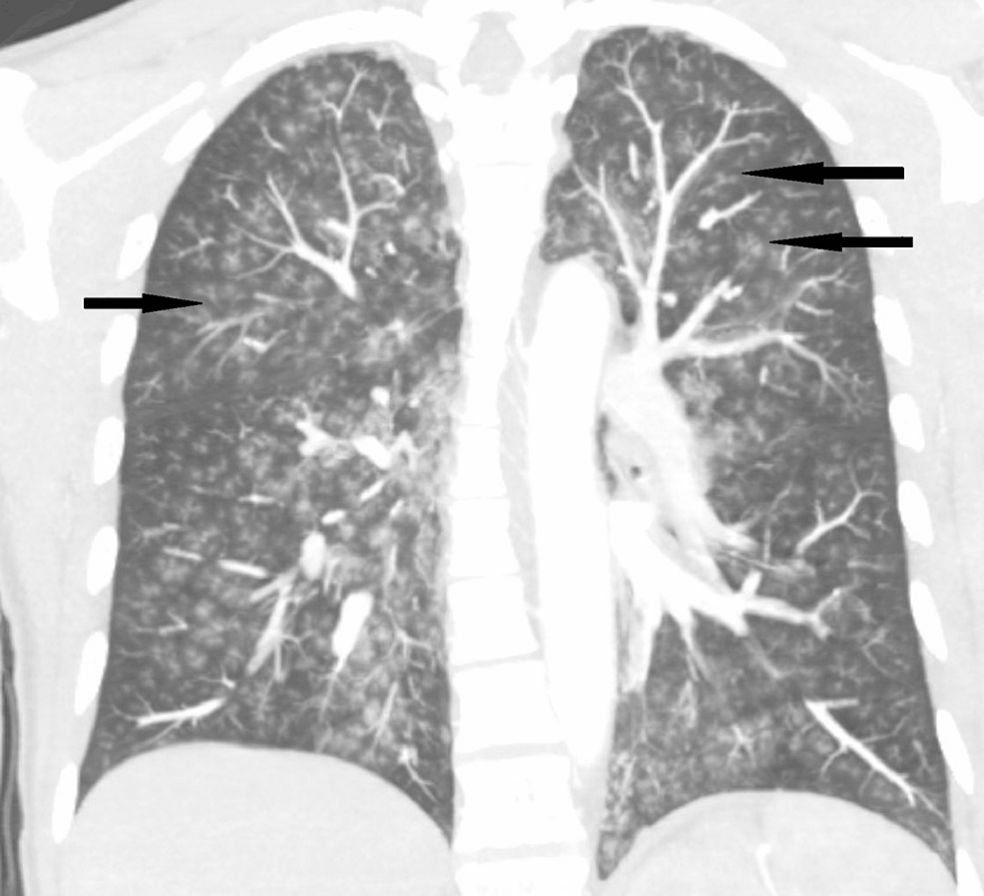
Coronal view of CT angiogram of the chest showing bilateral extensive nodular opacities (examples shown by arrows)

She was started on supplemental oxygen via a high-flow nasal cannula for respiratory support due to increased work of breathing. Complete blood count, complete metabolic panel, lactic acid, troponin, creatine phosphokinase, urinalysis, urine pregnancy test, and N-terminal pro B-type natriuretic peptide (NT-pro-BNP) were unremarkable. Urine drug screen was negative. Alcohol level was undetectable. Computerized tomography (CT) angiogram of the chest showed no evidence of pulmonary emboli. It showed bilateral extensive nodular ground-glass opacities (Figure 1).

**Figure 2:**
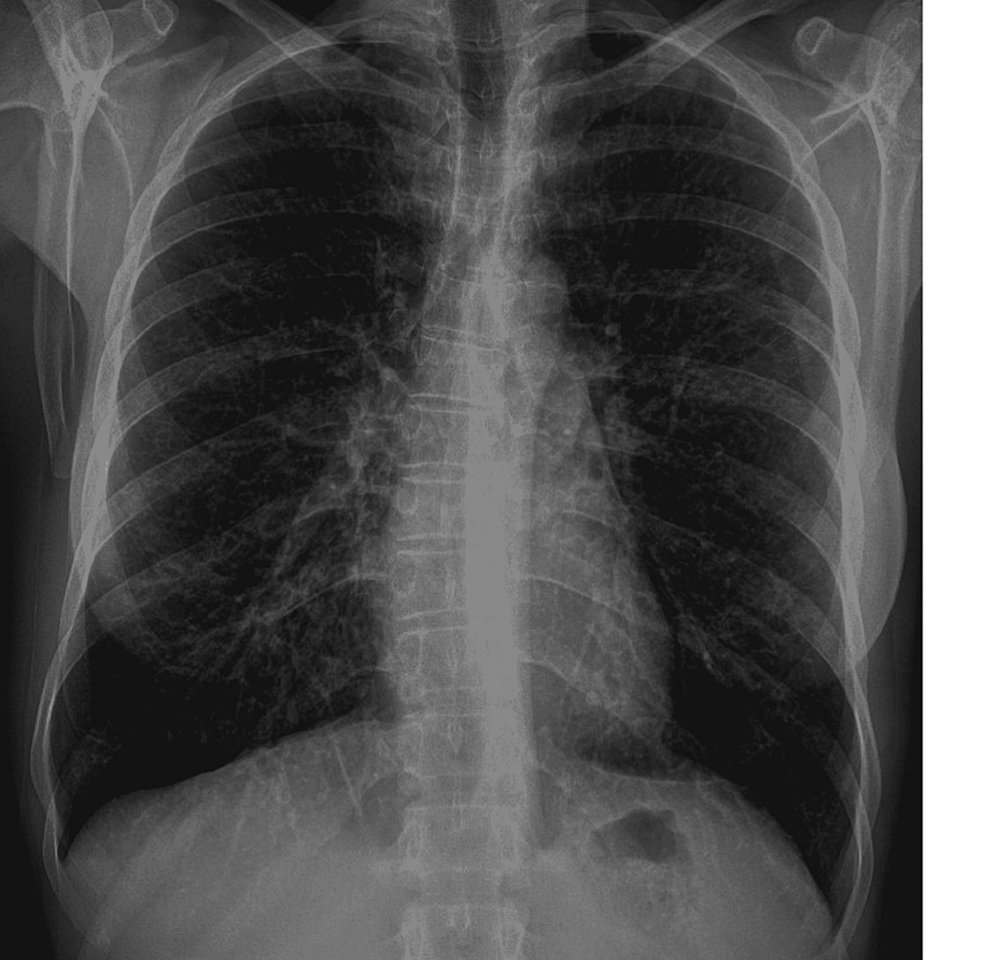
Follow-up chest radiograph after two weeks showing complete resolution of radiological abnormalities (normalized).

She was admitted to the intensive care unit (ICU), continued on respiratory support, and started on broad spectrum antibiotic coverage with vancomycin, piperacillin-tazobactam, and azithromycin due to diffuse infiltrates. Erythrocyte sedimentation rate (ESR) was 2 mm/hr, C-reactive protein (CRP) was 0.5 mg/dl, respiratory viral panel was negative, procalcitonin was elevated at 2.52 ng/m, and echocardiogram was unremarkable. By the next day, the patient had a clinical decline from a respiratory standpoint and was transitioned to bilevel positive airway pressure support (BiPAP). Due to CT findings of bilateral opacities, history of e-cigarette use, and worsening respiratory status despite antibiotic therapy, EVALI was suspected, and the patient was started on intravenous methylprednisolone 40 mg twice daily. The patient’s respiratory status improved in the next 24 hours, she was weaned off respiratory support, and saturation was maintained at >90% on room air. Urine culture resulted as >100,000 colonies of *Escherichia coli* *(E. coli)*. With the rapid improvement in respiratory status and an alternate source of infection, EVALI was thought to be the most plausible explanation for the lung findings. Complete cessation of any inhalational products was advised. The patient was discharged on a seven days taper of oral methylprednisolone. On outpatient follow-up in two weeks, a chest radiograph showed complete resolution of pulmonary abnormalities (Figure 2).

## Discussion

Since originally being identified in 2019, with approximately 80% of diagnosed individuals being under the age of 35 and maintaining significant morbidity and mortality, the necessity to understand the mechanism of EVALI has continued to rise [7, 8]. Several theories have since emerged with an emphasis on the causal effects of vitamin E acetate within e-cigarettes and the subsequent alteration of lipids within lung macrophages and alveolar type II cells (ATII) [9].

Product sampling by the United States Food and Drug Administration (US-FDA) and geographically diverse state laboratories, in addition to patient fluid samples from the CDC have indicated the presence of vitamin E acetate (VEA, also known as α-tocopherol acetate) is strongly linked to the majority of EVALI cases [6, 7]. Simultaneously, other chemicals found within e-cigarettes cannot be ruled out at this time due to a lack of sufficient evidence [7]. VEA is utilized to dilute THC concentrations within the liquid consumed for e-cigarettes [3, 10]. As the product is vaped, VEA will noncovalently bond with THC, causing the simultaneous release of a toxic ketene (R’R”C=C=O), and allows the complex to enter the lungs [11, 12]. The lipo-hydrophilic chemical properties allow VEA to then settle on the lungs’ surfactant layers to alter phospholipids into a liquid crystalline state which loses the surfactant’s ability to decrease alveolar surface tension [10].

The most common presentation is respiratory symptoms such as cough (80%), dyspnea (86%), and hypoxemia (77%) alongside 86% with gastrointestinal complaints of nausea (71%) and vomiting (70%) [11]. Constitutional symptoms are also common (94%) with subjective fever (79%), chills (50%) and fatigue (47%) [11, 13]. We suspect our patient had a cardiac arrest due to severe hypoxemia. An echocardiogram ruled out hypertrophic obstructive cardiomyopathy (HOCM). No arrhythmia was noted during the hospital stay. She did not respond to empiric treatment of bacterial infections. Commercially available comprehensive viral panel was negative. 

With the novelty of EVALI and the majority of currently published studies focused on case series and observational studies, there are limitations on the optimal treatment for EVALI. One study focused on 98 patients in Illinois and Wisconsin indicated the necessity of supplemental oxygen in 76% of patients; 22% needed non-invasive ventilation, and 26% required intubation and mechanical ventilation [13]. Additional documentation indicated that 92% of these patients received systemic glucocorticoids and a subsequent improvement of symptoms in 65% of those patients [13, 14]. This is supported by the CDC treatment framework indicating that glucocorticoids may provide symptom relief with a cautious caveat added given the limited studies [7]. The improvement of symptoms with the use of glucocorticoids suggests inflammatory pathways being activated in the patients of EVALI [14]. Separately, the CDC’s current framework outlines the necessity of patients eligible for outpatient management to maintain normal oxygen saturation without respiratory distress, have no comorbidities acting on pulmonary reserves, and have reliable access to healthcare with a strong support system [7]. Patients hospitalized should be documented as clinically stable for a minimum of 24 hours prior to discharge, followed by long-term follow-up for one to two months utilizing spirometry, diffusion capacity of lung for carbon monoxide (DLCO), and chest radiographs [7]. Both outpatient and inpatient management should include cessation of e-cigarettes and/or vaping, which may provide full resolution of symptoms in patients with mild cases of EVALI [15, 16]. Our patient had complete resolution of symptoms and imaging abnormalities with e-cigarette cessation and steroid therapy.

The abruptness of EVALI emerging as an outbreak in 2019 throughout the United States prompted state and federal health agencies to deploy surveillance and disease monitoring [12]. A six-month period between September 2019 and February 2020 showed numbers of cases moving from a maximum to a continuous decline, with a total of 2807 cases reported. Further updates of the number of cases were halted after February 2020 by the CDC due to both declining incidence and the emergence of the current COVID-19 pandemic which has made it difficult to analyze the effect of community efforts [17]. The continuing community efforts to promote smoking cessation have indirectly increased the marketing and usage of electronic devices, such as e-cigarettes, as a “safer” alternative to conventional smoking tobacco products [18]. The result of this marketing has seen the global e-cigarette/vaping market be valued at approximately $14 billion dollars in 2018 and is expected to double through 2022 [19]. Despite the constraints placed on the limited understanding of the mechanism behind EVALI and optimal treatment plans, current community and global efforts have been focused on education and stricter regulations. The success of stricter regulations can be adapted from Australia and the United Kingdom, where nicotine-containing e-cigarettes are banned, and have demonstrated improved smoking cessation strategies through tobacco control rather than the promotion of e-cigarette utilization as an alternative [18, 20].

## Conclusions

EVALI should be considered a confounding cause of acute cardio-pulmonary decline in patients with a history of e-cigarette/vaping use, even if they do not present with classic respiratory symptoms. Severe hypoxemia may lead to other complications in a patient, like cardiac arrest in our patient. More studies are required to determine if the treatment should be co-administered rather than waiting for other conditions to be ruled out first or treatments to fail. Our patient presented with non-typical symptoms of EVALI, and the diagnosis was not considered till later, based on assumptions that all other possibilities need to be ruled out first. Although the use of e-cigarettes/vaping products is declining based on public health data, there is still a large number of users in the community, and the history of usage should be proactively considered. 
